# Systematic enhancement of functioning as a therapeutic technique in conversion disorder

**DOI:** 10.4103/0019-5545.49454

**Published:** 2009

**Authors:** Chittaranjan Andrade, Savita G. Bhakta, Nagendra M. Singh

**Affiliations:** Department of Psychopharmacology, National Institute of Mental Health and Neurosciences, Bangalore - 560 029, India

**Keywords:** Aphonia, conversion disorder, dumbness, hysteria, paraplegia, systematic enhancement of functioning, symptom removal in conversion disorder

## Abstract

To explicitly outline a therapeutic technique for symptom removal in conversion disorder. We describe one patient with conversion dumbness and another with conversion paraplegia. The first patient was successfully treated in a single session, and the second was successfully treated across two weeks, both using systematic enhancement of functioning as a technique for symptom removal. This technique encourages the patient to express the desired behavior to whatever extent possible; subsequently, the patient is encouraged to gradually amplify the response until normal levels of functioning are achieved. The technique outlined is simple and practical but nevertheless receives no mention in conversion disorder literature. The technique can be applied to any situation in which behavioral amplification is desired.

## INTRODUCTION

Standard texts describe diverse methods of treatment of patients with conversion disorder. These include the identification and removal of the stressor (wherever possible), improvement of coping strategies, abreaction, provision of emotional support, reduction of anxiety levels, and provision of insight. Techniques more specifically oriented towards symptom removal include the use of reassurance, placebo, suggestion, hypnosis, and prayer, along with a removal of secondary gain.[1,2] In some situations, specific behavioral techniques, such as aversion, may be helpful.[3]

We present two cases of conversion disorder in which physiological functioning was restored by encouraging the patient to express the affected function, and to subsequently gradually amplify the response until normal functioning was recovered. To the best of our knowledge, this procedure, which we refer to as systematic enhancement of functioning, has not been explicitly detailed in the literature on the management of conversion disorder.

## CASE REPORTS

### Case 1

Mr. A, a 50-year-old petty businessman from Bangalore, was caught in a riot during a visit to another city. His valuables were taken away from him, his clothes were torn, and he was beaten up; he managed to escape, but had to live in the gutters and eat from dustbins to survive. He witnessed horrific examples of mob violence during this period. Over a period of several days, he managed to travel a distance of over a thousand kilometers to return to his family. He had to journey ticketless by various forms of public transport, and beg for food and water. When he returned home, he was altogether unable to speak.

He was brought for consultation several days later because of his inability to speak. At the time of examination, he was able to vocalize but not verbalize. His affect was markedly anxious. A diagnosis of conversion disorder with motor deficit (DSM-IV 300.11) was made.

Reassurance was provided to the patient that his symptom was benign and curable. Symptom removal was initiated through systematic enhancement of the character of his vocalizations.

He was encouraged to repeatedly vocalize "aaaaaaaaaa…." He was able to do this without difficulty. He was next instructed to make sounds with his lips together; thus, "mmmmmmmmm…." He complied successfully. Then, he was asked to put the two sounds together as "mmmmmmm… aaaaaaaa… mmmmmmmm… aaaaaaaa." Afterwards, he was asked to progressively shorten each sound, and the intervals between the sounds, until he was articulating "mmaaa, mmaa, mmaa…."

The syllable ‘ma’ represents ‘mother’ in several Indian languages as well as in English. It was suggested to him that his articulation was a favorable omen, and his progress was applauded. “You were unable to speak, and now, look! You are already saying ‘mother’.“

Using a similar procedure, he was helped to progress to other simple combinations of vowel and consonant sounds such as ‘na’, ‘ha’, ‘sa’, ‘moo’, etc. The next step was the combination of sounds to form meaningless words such as ‘mana’, ‘nama’, ‘saha’, etc. Eventually, he was able to produce meaningful words, and then formal speech in a soft and hesitant but clearly audible fashion.

Before the end of this approximately 90-minute session, he was encouraged to ventilate about his experiences. He was provided with reassurance, family and social support were recruited, and an anxiolytic was prescribed for a brief period. Two weeks later, he was speaking normally.

### Case 2

Mr. N, a 26-year-old farmer, was brought with a one-day history of loss of strength in his lower limbs. The previous day, hostile farmers from a nearby village had raided his fields and destroyed his crops while his family was away; instead of defending his land, he had hidden in his house. When his family returned, he was discovered lying in his bed, unable to move his lower limbs.

On examination, he was found to have no apparent power in any muscle group in his lower limbs. Superficial and deep reflexes, however, were normal. A diagnosis of conversion disorder with motor deficit (DSM-IV 300.11) was made. Over the subsequent week, attempts were made to provide insight into the nature and cause of the symptom, and to effect symptom removal through the use of reassurance, placebo, suggestion, electrical stimulation of the muscles of the lower limbs, removal of secondary gain, and other methods; none proved successful.

Systematic enhancement of functioning was then attempted. He was encouraged to move the great toe on one foot, and was lavishly praised when, after much time and effort, a flicker of movement was elicited. This movement was practiced until he was able to move the toe consistently. He was then encouraged to move the great toe of the other foot. Afterwards, he was encouraged to dorsiflex and plantarflex each foot, then swing his legs at the knees as they dangled freely over the edge of a chair, and so on, until he was able to perform most movements of most muscle groups in the lower limbs.

He was subsequently encouraged to walk. Initially, he was only able to make stepping movements while supported by a ward boy on each side; however, he gradually progressed to weight-bearing with support from the ward staff, and then weight-bearing with support from furniture and railings. He eventually walked on his own, hesitantly, but without support. Successful restitution of functioning through systematic enhancement took about 2 weeks.

## DISCUSSION

In general terms, symptom removal was effected by speech therapy[4] in the case of Mr. A and by physiotherapy[5] in the case of Mr. N; to this extent, the techniques applied are not new. Nevertheless, we seek to record the principle employed as an explicit therapeutic technique because, to the best of our knowledge, systematic enhancement of functioning as a method to treat conversion symptoms has not been explicitly described in standard Western texts[1,2] or even standard texts in third world countries, such as India, in which conversion disorder is more common.[6,7] In behavioral terms, systematic enhancement as described in this study is similar to successive approximations, or shaping, both of which concepts are familiar to behaviorists.[8]

Why do we lay emphasis on the inclusion of this technique in the outline of the management of conversion disorder? Unless a therapeutic technique is explicitly described, it is unlikely that it will be consistently adopted in clinical practice. As a result, some patients may not receive the best possible treatment package, and symptom removal may not be easily or completely effected. Consider, for example, that Mr. N. did not regain the use of his limbs with the usual methods of treatment but eventually recovered only with the use of systematic enhancement. And, in this context, it should also be recalled that, in conversion disorder, if symptom removal is not effected early, chronicity may lead to refractoriness and the establishment of the symptom.[1]

We suggest that systematic enhancement of functioning as a technique for symptom removal can be used alone or in combination with techniques that are based on placebo or suggestion. It is likely, however, that systematic enhancement will be effective in only those patients whose loss of function is not complete, or to those who are recovering function as a result of the use of other techniques of symptom removal.

As a final note, with reference to our first case, some attention must be paid to terminology. Aphonia refers to the inability to produce sound; yet, several reports on hysterical aphonia describe methods of sound production in the treatment of the patient. For example, McLeod and Hemsley[9] used visual feedback of vocal intensity in the treatment of one patient with hysterical aphonia. Harris and Richards[4] described speech therapy as a means of symptom removal in 14 young aphonics. Clearly, the patients in the two reports could not have been aphonic. It is therefore better to use conversion dumbness as a descriptor of a hysterical inability to articulate.
